# Reoperation following Pancreaticoduodenectomy

**DOI:** 10.1155/2012/218248

**Published:** 2012-09-12

**Authors:** J. R. Reddy, R. Saxena, R. K. Singh, B. Pottakkat, A. Prakash, A. Behari, A. K. Gupta, V. K. Kapoor

**Affiliations:** Department of Surgical Gastroenterology, Sanjay Gandhi Postgraduate Institute of Medical Sciences (SGPGIMS), Rae Bareily Road, Lucknow 226014, India

## Abstract

*Introduction*. The literature on reoperation following pancreaticoduodenectomy is sparse and does not address all concerns. *Aim*. To analyze the incidence, causes, and outcome of patients undergoing reoperations following pancreaticoduodenectomy. *Methods*. Retrospective analysis of 520 consecutive patients undergoing pancreaticoduodenectomy from May 1989 to September 2010. *Results*. 96 patients (18.5%) were reoperated; 72 were early, 18 were late, and 6 underwent both early and late reoperations. Indications for early reoperation were post pancreatectomy hemorrhage in 53 (68%), pancreatico-enteric anastomotic leak in 10 (13%), hepaticojejunostomy leak in 3 (3.8%), duodenojejunostomy leak in 4 (5%), intestinal obstruction in 1 (1.2%) and miscellaneous causes in 7 (9%). Patients reoperated early did not fare poorly on long-term follow up. Indications for late reoperations were complications of index surgery (*n* = 12), recurrence of the primary disease (*n* = 8), complications of adjuvant radiotherapy (*n* = 3), and gastrointestinal bleed (*n* = 1). The median survival of 16 patients reoperated late without recurrent disease was 49 months. *Conclusion*. Early reoperations following pancreaticoduodenectomy, commonly for post pancreatectomy hemorrhage, carries a high mortality due to associated sepsis, but has no impact on long-term survival. Long-term complications related to pancreaticoduodenectomy and adjuvant radiotherapy can be managed successfully with good results.

## 1. Introduction

Descriptions of post pancreaticoduodenectomy (PD) reoperations have largely addressed relaparotomy for early complications such as postpancreatectomy hemorrhage (PPH) and pancreaticoenteric anastomotic leak (PEA) with associated intraabdominal collection [1, 2]. The literature on other indications is very limited. Quite a number of studies have addressed the long-term survival of patients undergoing PD and the need for readmission in them on long-term follow up [3, 4]. However, there is very limited data that specifically addresses the need for and the outcome of surgical reintervention in these patients on long-term follow up. The aim of this study was to analyze the following.

- The incidence and causes of early and late reoperations following PD.
- Factors predicting the need for early reoperation and its related mortality.
- The outcome of patients undergoing early and late reoperations.

## 2. Patients and Methods

Five hundred and twenty patients underwent PD between May 1989 and September 2010 at the Department of Surgical Gastroenterology, Sanjay Gandhi Postgraduate Institute of Medical Sciences, a tertiary referral institute in the northern part of India. Data was retrieved from a prospectively maintained database which included variables recorded during the index hospitalization and further readmissions if any. Information about patient follow up was obtained from follow up cards and telephonic follow up interviews. 

All pancreaticoduodenectomies at our institute were performed by or under the direct supervision of consultant surgeons. Preoperatively all these patients underwent routine blood counts, liver and renal function tests, abdominal sonography, and an abdominal computed tomography (CT) scan for tumour staging. A side viewing endoscopic examination with biopsy was contemplated in almost all patients as a predominant number of patients who undergo PD at our hospital have periampullary carcinoma. In patients with a negative biopsy and a demonstrable CT scan evidence of a periampullary mass, decision to proceed with PD was taken. Endoscopic ultrasound was selectively used in those patients with a negative biopsy and no evidence of a mass lesion on CT scan. A preoperative endoscopic biliary drainage procedure with stenting was carried out in those patients with cholangitis, high preoperative bilirubin (>15 mg/dL), or poor nutritional status and surgery was then performed 4–6 weeks after stenting. All patients received preoperative antibiotic dose of cefoperazone and sulbactam 2 g and amikacin 500–750 mg at the time of induction. An equal number of patients underwent a pylorus preserving PD or a classical Whipple procedure according to the surgeon's preference. Pancreatic reconstruction was performed first by an end to end or end to side pancreatico-jejunostomy in 514 patients. Of the remaining, 3 patients underwent pancreatico-gastrostomy and 3 had no pancreatico-enteric reconstruction due to underlying acute pancreatitis and necrosis. Duct to mucosa and pancreatic dunking or invagination was performed equally based on surgeon preference and pancreatic duct stenting was used selectively. This was followed by an end to side hepaticojejunostomy and antecolic duodenojejunostomy or gastrojejunostomy. Nasojejunal tube was used preferentially over feeding jejunostomy as a feeding access. Intraoperative octreotide (100 ug stat) was used selectively in those patients with a soft pancreas and continued for 5 days postoperatively. Abdomen was closed with drainage. A nasogastric tube was placed for gastric decompression. Postoperatively drain fluid and serum amylase levels were estimated on postoperative days 4 and 7.

Reoperations were classified into early and late. Reoperations performed during index hospital admission following PD were classified as early while those reoperations performed any time after the index hospitalization were classified as late. Patients operated for indications unrelated to complications of index surgery (PD), tumour recurrence, or adjuvant radiotherapy were excluded. The reoperation data was retrieved from the database. Patients requiring early reoperations due to complications of index surgery were compared with those who did not need reoperation. The parameters evaluated were demographic factors, clinical presenting symptoms, intra-operative parameters, pathology, and postoperative complications. A univariate analysis was done to determine factors predictive of early reoperation. Chi-square test was used for categorical variables and Mann-Whitney *U* test for continuous variables. A multivariate logistic regression analysis was done to identify the variables independently predicting reoperation within this group. A similar analysis was done to identify the factors predictive of in-hospital mortality in patients undergoing early reoperation. Patients requiring late reoperations were classified into four groups: reoperation for complications of index surgery (group 1), tumour recurrence (group 2), complications of radiotherapy (group 3), and miscellaneous indications (group 4). To analyze the impact of early reoperations on survival, a Kaplan Meier survival curve was constructed including patients undergoing pancreatico-duodenectomy till September 2007, and statistical significance was tested using log-rank test. Median survival of patients undergoing late reoperation was analyzed after excluding patients being reoperated for tumour recurrence. *P*  value < 0.05 was considered as significant. Statistical analysis was performed using SPSS version 15 (SPSS, Chicago, IL, USA).

## 3. Results

Between May 1989 and September 2010, 520 patients underwent PD. Of these, 26 patients (5%) underwent PD for benign disease and 494 patients (95%) for malignant disease. The median age was 52 years (range 14–82 years). The in-hospital mortality rate was 8.1 percent (42 of 520), the overall morbidity rate was 62% per cent (322 of 520), and the median hospital stay was 14 days (range 5–112 days). 96 of these 520 patients (18.5%) were reoperated upon. 72 (75%) of these were early, 18 (18.8%) were late reoperations, and 6 patients (6.2%) had both early and late reoperations. For the purpose of analysis, the 6 patients who underwent both early and late reoperation were included in both the groups, thereby accounting for 78 patients (72 + 6) who underwent early reoperation and 24 patients (18 + 6) who underwent late reoperation. Among patients undergoing early reoperations, there were 53 males and 25 females with a median age of 52 years (range 23–72 years). Median time to reoperation was 8 days (range 0–59 days), 42% of patients were reoperated within 5 days, 63% within 10 days, and 89% within 20 days following PD.

### 3.1. Early Reoperations

**Figure 1:**
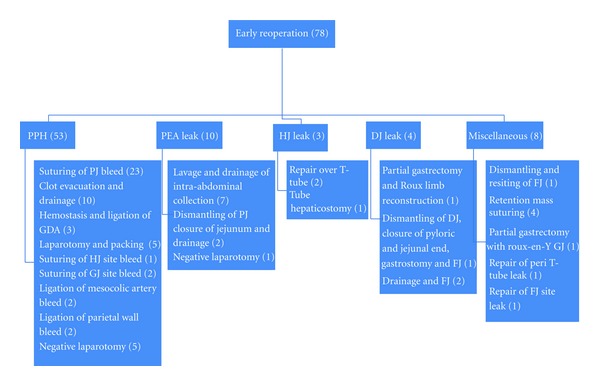
Indications of early reoperations and surgeries performed.

The indications for early reoperation were postpancreatectomy hemorrhage (PPH) in 53 patients (68%), pancreatico-enteric anastomotic leak (PEA) with intra-abdominal collection in 10 (13%), hepatico-jejunostomy (HJ) leak in 3 (3.8%), duodeno-jejunostomy (DJ) leak in 4 (5%), intestinal obstruction in 1 (1.2%), and miscellaneous causes in 7 (9%) such as wound dehiscence (*n* = 4), feeding jejunostomy or T-tube related complications (*n* = 2), and afferent loop obstruction (*n* = 1). 70 patients were reoperated once, 7 patients twice and 1 patient thrice. The surgical indications and the interventions performed are enumerated in Figure 1.

Of the 53 patients undergoing reoperation for PPH, 41 patients (77.3%) had late bleeds (>24 hours) and 32 patients (60.4%) had extra luminal bleeds. The commonest surgery for PPH was suture ligation of the pancreatic cut surface bleeder which was done in 23 (43%) patients. Nearly 10% of patients operated for PPH had a negative laparotomy as no active source of bleed was identified. The median time to surgery in patients being reoperated for PPH was 5 days (range 0–59 days). 10 of these 53 patients (18.8%) presented with rebleed following first relaparotomy. Among these 10 patients, 4 patients required a second relaparotomy and in 6 patients angiographic embolization was done for gastroduodenal artery (*n* = 4) or right hepatic artery pseudoaneurysm (*n* = 2). The 10 patients reoperated for PEA leak with intra-abdominal collection were done so due to failure of percutaneous drainage or lack of radiological access for the same. 3 patients were reoperated for HJ leak, of which 2 were within 48–72 hours due to right subhepatic drain showing bile effluent and a third patient was reoperated on postoperative day 24 for persistent bile leak for which a tube hepaticostomy was done. Median postoperative stay following index surgery was significantly longer in patients undergoing early reoperation (25.5 days versus 13 days; *P* = 0.000). The in-hospital mortality was also significantly more in patients undergoing early reoperation (33.3% versus 3.6%; *P* = 0.000). Over the years, the number of pancreaticoduodenectomies performed at our institute has significantly increased. With experience thereby, there has been a reduction in our overall in-hospital mortality rate, incidence of PPH, reoperation rate, and mortality rate following reoperation and this is enumerated in Table 1.

Factors predictive of early reoperation on univariate analysis were preoperative factors such as longer duration of jaundice (>3 months) (*P* = 0.051) and total bilirubin > 10 mg% (*P* = 0.010), intra-operative parameters such as blood loss (*P* = 0.001) and requirement of intra-operative blood transfusion (*P* = 0.010), and occurrence of post-operative complications in the form of PPH (*P* = 0.000), PEA leak (*P* = 0.001), HJ leak (*P* = 0.000), DJ leak (*P* = 0.000), intra-abdominal collection (*P* = 0.000), delayed gastric emptying (DGE) (*P* = 0.007), acute renal failure (*P* = 0.000), and septicemia (*P* = 0.000)Table 2.

On multivariate analysis using the logistic regression model, preoperative duration of jaundice > 3 months (*P* = 0.019), occurrence of postoperative complications such as PPH (*P* = 0.000), intra-abdominal collection (*P* = 0.027), and DJ/GJ leak (*P* = 0.041) were independently predictive of the need for early reoperations (Table 3). 

Of the 26 patients who had postoperative mortality following early reoperation, the underlying cause was PPH in 17 patients, PEA leak and intra-abdominal collection in 6 patients, DJ leak, feeding jejunostomy site leak with peritonitis and acute renal failure in 1 patient each. In the 17 patients who expired following reoperation for PPH, 15 (88%) of them were reoperated for late bleeds (>24 hours following PD). Septic shock with supervening multiorgan failure was the main cause of death in all these patients. Analysis of factors affecting mortality in patients undergoing early reoperation showed that the only significant factor on multivariate analysis was development of postoperative acute renal failure (*P* = 0.014; Exp. (*B*) = 0.109, 95% CI = 0.020–0.596) defined as an increase in serum creatinine level × 1.5 of the patients baseline or urine output <0.5 mL/kg/h for at least 6 hours.

### 3.2. Late Reoperations

Twenty four patients underwent late reoperations (16 males; 8 females, median age 47.5 years (range 20–68 years)). Of these 6 patients had also undergone early reoperations, 4 for PPH and 2 for PEA leak with intra-abdominal collection. The indications for late reoperations were complications of index surgery (group 1) (*n* = 12), recurrence of primary disease (group 2) (*n* = 8), complications related to adjuvant radiotherapy (group 3), (*n* = 3) and miscellaneous causes (group 4) (*n* = 1)Table 4. Among the 12 patients in group 1, the predominant number were incisional hernias (4 patients), 2 patients presented with symptoms suggestive post-PD chronic pancreatitis and required a revision pancreatico-enteric anastamosis and are symptom free on long-term follow up. In group 2, 4 patients presented with peritoneal dissemination with subacute intestinal obstruction and of these 2 patients were amenable for a bypass procedure to relieve obstruction. 2 patients with duodenal gastro-intestinal stromal tumours on long-term follow up presented with liver metastasis, in spite of continued imatinib therapy and required a right hepatectomy and a nonanatomic liver resection, respectively. The patient who underwent right hepatectomy expired 5 months later due to extensive metastasis in the remnant liver and the other is alive and disease free 29 months following nonanatomic resection of liver metastasis. One patient in the disease recurrence group presented with intraabdominal hemorrhage following intraperitoneal rupture of liver metastasis and expired in the postoperative period. In group 3, two patients were reoperated for radiation enteritis induced jejunal stricture and DJ stricture and one patient required an emergency relaparotomy for a colonic and afferent jejunal limb necrosis. The median time to reoperation was 21 months (range 5–128 months) and the median hospital stay was 13 days.

**Figure 2:**
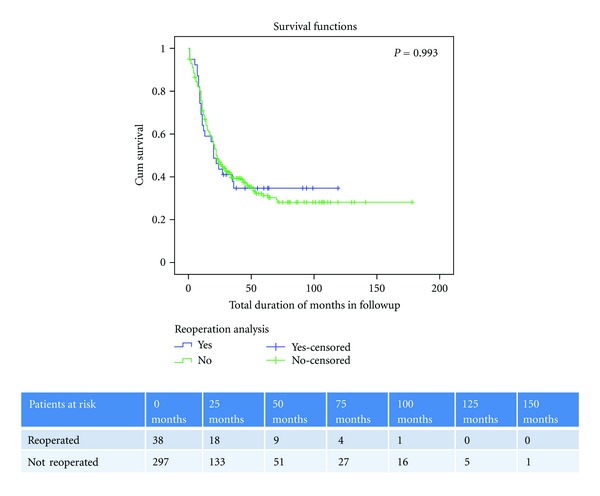
Kaplan Meier survival curve. Comparison of 3 year survival of patients undergoing early reoperation versus those patients not requiring reoperation.

To analyze the impact of reoperation on survival, patients with a minimum follow up of 3 years were included and Kaplan Meier survival curves were generated. 3 year survival of patients undergoing early reoperations (*n* = 38) was compared to those patients not requiring reoperation (*n* = 297). The median survival of patients undergoing reoperation was 20 months and that of those not undergoing reoperation was 23 months. There was no statistical difference in survival between the two groups *P* = 0.993 on log rank analysis (Figure 2). In the late reoperation group (*n* = 24), excluding the 8 patients reoperated for disease recurrence the median survival was 49 months.

## 4. Discussion

Experience from high volume tertiary care centers around the world has shown a significant decrease in mortality following PD over the last couple of decades. Despite a significant decrease in postoperative mortality, PD is still associated with a fairly high postoperative morbidity as reported by various centers in the range of 30–60% [5, 6]. Some of these common postoperative complications including PPH and PEA leak with intra-abdominal collection and associated septic complications may require surgical intervention, despite the widespread availability of endovascular and radiological interventions. This morbidity prolongs hospital stay and results in mortality in a significant proportion of patients. Reoperative surgery after PD is a difficult undertaking and the reoperation itself may be the cause of further morbidity and mortality [7]. Due to increasing long-term survival of patients undergoing PD for periampullary carcinoma, some may on long-term follow up develop complications that may need intervention. These complications may be related to the complications of primary surgical procedure, recurrence of the primary disease per se, and/or complications of adjuvant radiotherapy.

Reoperation rates in series dealing with pancreatic head resection have varied from 4–11% [7]. Recent studies by Standop et al. and Shukla et al. dealing specifically with operative reinterventions have also shown similar rates [7, 8]. In the present series, we had an overall reoperation rate of 18.5%. With increasing experience and better access to interventional radiological expertise, our reoperation rate has significantly decreased to 7.4% over the last 5 years.

PPH is one of the grave complications following PD and occurs in 2–20% of patients as reported by various series [9–11]. Our overall incidence of PPH following PD is 18% but this has decreased to 13% over the past 5 years. Although an uncommon occurrence, hemorrhage following PD has been associated with high mortality ranging from 14% to 56% [12–14]. Indeed, hemorrhage is an important predictor of prognosis and mortality in patients with PEA leak. According to the International Study Group of Pancreatic Surgery classification, the management of PPH depends on various factors like time of onset of bleed (early versus late), location (intraluminal versus extra-luminal), and severity of bleed (mild versus severe) [15].

PPH was the predominant cause of early reoperation in our subset of patients similar to that reported by the other series [7, 8]. Overall 56% (53 of 94) of our patients with PPH underwent relaparotomy for the same. The number of reoperations for PPH in our series has reduced by 25% (from 65% to 40%) in the past 5 years due to better use of endovascular coil embolization. Our higher overall reoperation rate for PPH is due to the fact that all patients with early PPH and hemodynamic instability undergo relaparotomy and lower threshold for reoperation in delayed presentation of PPH in event of delay in the interventional radiology back up because of pre-occupation. The aggressive use of surgical intervention for management of delayed presentation of PPH in our study is similar to that reported by de Castro et al. [9], who preferred surgical intervention over embolization for management for the delayed massive hemorrhage following major pancreatic or biliary surgery. The basic principles of relaparotomy surgery are hemostasis and wide drainage. One of the important causes of hemorrhage in our experience as previously published and also in the present series has been a bleeding pancreatic stump following an intact or a disrupted pancreatic anastomosis [13]. Bleeding from the pancreatic stump can present as intra-abdominal bleed manifesting through the intra-abdominal drains due to secondary disruption of the PEA or can present as intraluminal bleed in the form of hematemesis or hemorrhagic aspirate in the nasogastric tube. This can be localized preoperatively by evidence of brisk bleeding from afferent loop of jejunum on endoscopy. A significant proportion of our patients who present with an intraluminal bleed have a pancreatic cut surface bleeder and hence are not amenable for endoscopic management thereby requiring relaparotomy. The management options are variable depending upon whether the PEA is intact or not. In a wide anastomotic disruption with a bleeding pancreatic stump, direct suturing should be done to achieve hemostasis. In an occasional patient without significant disruption of the anastomosis serial jejunal clamping may indicate the segment of the bowel from which the bleeding is originating and hemostasis can then be achieved by suturing through an appropriately placed enterotomy [16]. If at surgery the site of hemorrhage is not found and the stumps of ligated vessels have been carefully inspected, then enterotomy should be considered to look at one or more of the several suture lines, which may be the source of bleeding. In addition to local hemostasis, it is of utmost importance to drain all the adjacent collections and abscesses, as these may lead to further episodes of rebleed due to erosion of adjacent vascular structures. It may not always be possible to achieve adequate surgical control due to diffuse nature of the ooze from the operative site raw area or retroperitoneum. In such cases, abdominal packing can be done as temporizing measure and planned relaparotomies can be done once the patient has stabilized and the coagulation abnormalities have been corrected. The problems encountered with surgery for rebleed are two-fold; first intraoperatively it may not be always possible to identify the site of bleed due to difficult access in the presence of dense adhesion, friable, and inflamed tissues and secondly a small percentage of patients may require a second relaparotomy or a prophylactic embolization of the common hepatic or splenic artery after surgery to prevent the occurrence of rebleed [10, 13].

The second commonest cause of reoperation after PPH was PEA leak with intra-abdominal collection. Majority of these collections can be managed successfully by percutaneously placed drains under image guidance [17]. Relaparotomy is occasionally required in some patients due to failure of percutaneous drainage or lack of radiological access for the same in the presence of persisting sepsis. Disrupted PEA can be dealt with in one of the following ways. Resuturing has been attempted by some but it invariably fails in the presence of edematous friable tissues. Others, including us, are in favour of dismantling the anastamosis completely, closing the jejunal loop and providing drainage of the pancreatic duct, often with a laparostomy to ensure free drainage [13, 18]. This invariably leads to a pancreatic fistula but free drainage helps in controlling the sepsis. Still others have successfully opted for a completion total pancreatectomy to remove the focus of sepsis altogether [19]. Regardless of the surgical procedure chosen, it is of utmost importance to drain all collections and abscesses. Preoperative CT scan is a good guide to locate these collections in the presence of adjacent inflamed and friable tissues. 

Bilioenteric anastomotic leak is very uncommon following PD and the management is usually conservative [20]. Surgery is indicated in those patients presenting with bilious effluent in the drain early in the postoperative period, that is, within 48 hours. This is usually due to a technical problem and surgical correction of the same would lead to faster recovery and avoid the development of a strictured HJ. Deficiency of gastro-jejunostomy or duodeno-jejunostomy is very rare following PD. Some of these patients can present with an enterocutaneous fistula, which can be very difficult to manage due to the high output, persistent nature of the fistula, and associated pancreatic enzymatic leak. Revision surgery in the form of a revision roux- en-Y reconstruction or just a simple drainage with a feeding access can be done in these patients. Other infrequent indications of relaparotomy after PD are complete dehiscence of the laparotomy wound with evisceration which would need meticulous closure with interrupted sutures accompanied by retention mass sutures. Feeding jejunostomy related complications requiring relaparotomy were seen in a couple of patients in the present series. Albeit a simple procedure, FJ related complications can be catastrophic in some patients and hence the feeding access of choice in our patients is an intraoperatively placed nasojejunal tube.

Various surgical series have looked into the factors predictive of the occurrence of complications following PD. Some of them are presence of associated medical risk factors, need for preoperative biliary drainage, texture of remnant pancreas, and size of pancreatic duct [21–23]. The factors that were more frequently associated with early reoperation on multivariate analysis in the present study were longer preoperative duration of jaundice (>3 months), postoperative occurrence of complications such as PPH, presence of intra-abdominal collections, and leakage of alimentary tract reconstructions. Patients with longer duration of preoperative jaundice are more nutritionally depleted due to prolonged poor oral intake which thereby leads to increased postoperative complications [24].

Overall mortality following PD significantly increases after early reoperation, and is in the range of 13–60% as shown by Standop et al. [7], which included pooled data from various studies. Recent single institution series from Standop et al. [7] and Shukla et al. [8] dealing specifically with early reoperation have shown a significantly decreased mortality rate of 11.7% and 13%, respectively. Although our overall mortality rate is 33.3% following reoperation, this has decreased from 35.7% in the initial 16 years to 27.2% in the past 5 years. This can be attributed to the lower overall incidence of PPH, lesser number of patients being reoperated for PPH, and increased use of interventional radiology techniques for management of post-PD complications. Though non-surgical management options for PPH like endovascular coiling or stenting and endoscopic interventions cause lesser physiological insult, they have not been conclusively shown to be better than relaparotomy in terms of the success rate for management of delayed PPH [25, 26]. de Castro et al. [9] who favored surgical reintervention over embolization for the management of patients presenting with delayed hemorrhage following PD, reported an overall mortality of 22% but 5 of the 16 patients (31%) who underwent surgical re-intervention in their series expired in the postoperative period due to sepsis. A recent meta-analysis and a systematic review on the management of delayed PPH by Limongelli et al. [25] and Roulin et al. [26] have shown high mortality rates of 43% and 47%, respectively, following relaparotomy for PPH. This is similar to our study where the mortality rate following reoperation for late presentation of PPH was 36% (15 of 41). Roulin et al. [26] in their systematic review in addition showed that there was a statistically significant difference in favour of interventional radiology in term of mortality after PPH compared to relaparotomy. Although surgical intervention is very successful for the management of PPH, most of the patients presenting with late bleeds have underlying sepsis which contributes to the increased incidence of morbidity and mortality in them. 

Due to improved long-term survival of patients with nonpancreatic periampullary carcinoma as compared to pancreatic cancer, quite a few patients present on long-term follow up with complications related to the index surgery or disease recurrence which may be amenable to surgical intervention. With the increasing use of adjuvant radiotherapy to gain better local control and thereby improve disease-free survival, patients may require intervention for complications of the same. Excluding patients with malignant pancreatic neuroendocrine tumours, this issue has been very sparsely addressed in the literature with most of them being occasional case reports or short case series [27, 28]. 

The indications for late reoperations are varied. Patients undergoing second surgery for complications related to recurrence of primary disease following PD other than those with malignant neuroendocrine tumours usually have a relatively poor outcome even after resectional surgery for local or distant recurrences as shown by Nakano et al. and Fujii et al. [28, 29]. Excluding these patients, those presenting with late complications related to PD or complications of adjuvant radiotherapy can be managed successfully with good long-term outcome.

Early reoperation had no impact on 3 year survival of patients in our series. This is in contrast to that reported by Yeo et al. in the 1990s, in which they showed that in addition to the tumour pathologic characteristics, the addition of reoperation had a negative impact on long-term survival of patients undergoing PD for periampullary carcinoma [30]. The detrimental effect of postoperative complications on the long-term survival of patients undergoing PD for pancreatic cancer has been a matter of controversy [31, 32]. In the present study-patients surviving the initial insult of early reoperations had similar long-term survival rates to those who did not undergo an early reoperation.

## 5. Conclusion

In this study, 18.5% of patients were reoperated following PD. The two main indications for early reoperation were PPH (68%) and PEA leak with associated intra-abdominal collection (13%). Reoperation for PPH is usually indicated in patients with early presentation of PPH (<24 hours) or delayed presentation of PPH where angiographic embolization is not feasible or successful. Early reoperation when used judiciously in conjunction with arterial coil embolization continues to be an important tool in the armamentarium for the management of PPH in the present era. Although in-hospital mortality in this subset of patients was high (33.3%), this is largely due to the associated sepsis rather than insult of the reoperation. Early reoperation did not have a bearing on long-term survival (*P* = 0.993). On long-term follow up patients presenting with complications related to PD or adjuvant radiotherapy can be managed with good outcome.

## Figures and Tables

**Table 1:** Changes over time.

	1989–2000	2000–2005	2006–2010
Number of pancreaticoduodenectomies	160	132	228
Incidence of PPH	20% (32/160)	24.2% (32/132)	13.1% (30/228)
reoperation rate	17.5% (28/160)	21.2% (28/132)	9.6% (22/228)
Indications for reoperation			
(i) PPH	20	21	12
(ii) PJ leak with intra-abdominal collection	4	4	2
(iii) HJ leak		1	2
(iv) DJ/GJ leak	2		2
(v) Miscellaneous	2	2	4
Overall in-hospital mortality	11.9% (19/160)	9% (12/132)	4.8% (11/228)
In-hospital mortality following early reoperation	42.8% (12/28)	28.5% (8/28)	27.2% (6/22)
In-hospital mortality in patients not undergoing early reoperation	5.3% (7/132)	3.8% (4/104)	2.4% (5/206)

PPH: post pancreatectomy hemorrhage; PJ: pancreaticojejunostomy; HJ: hepaticojejunostomy; DJ: duodenojejunostomy; GJ: gastrojejunostomy.

**Table 2:** Univariate analysis of factors predicting the need for early reoperation.

Parameters	Early reoperations (*N* = 78)	Not reoperated early (*N* = 442)	*P* value
Age in years (median)	52	51.5	0.850
Gender (M/F)	53/25	318/124	0.498
Duration of jaundice > 3 months	21 (27%)	69 (15.6%)	0.051
Comorbidities	17 (21.8%)	111 (25.1%)	0.572
Preoperative hemoglobin (median)	11.0	11.2	0.425
Preoperative albumin (median)	3.5	3.5	0.643
Total bilirubin > 10 mg%	18 (23.1%)	51 (11.5%)	0.010
Preoperative biliary drainage	41 (52.6%)	276 (62.4%)	0.103
Duration of surgery (hours)	7.35	7.0	0.096
Blood loss (mL) (median)	750 mL	500 mL	0.001
Blood transfusion	50 (64.1%)	213 (48.2%)	0.010
	Malignancy (94%)	Malignancy (93%)	0.844
Pathology	Ampullary Ca (74%)	Ampullary Ca (71%)	
	Benign (6%)	Benign (7%)	
PPH	67.9%	9.3%	0.000
PEA leak	34.6%	14.3%	0.001
HJ leak	24.3%	6.3%	0.000
DJ/GJ leak	14.1%	2.5%	0.000
DGE	21.8%	10.2%	0.007
Intraabdominal collection	43.6%	10.2%	0.000
ARF	11.5%	1.6%	0.000
Septicemia	32%	6.3%	0.000
Postoperative hospital stay (Mean)	25.5 days	13 days	0.000
In-hospital mortality	26 (33.1%)	16 (3.6%)	0.000

PPH: post pancreatectomy hemorrhage; PEA: pancreaticoenteric anastamosis; HJ: hepaticojejunostomy; DJ: duodenojejunostomy; GJ: gastrojejunostomy; DGE: delayed gastric emptying; ARF: acute renal failure.

**Table 3:** Multivariate analysis of factors predicting the need for early reoperation.

Parameter	*P* value	Exp. (*B*)	95% CI for Exp. (*B*)
Duration of jaundice > 3 months	0.019	3.532	1.23–10.147
PPH	0.000	0.101	0.052–0.198
Intraabdominal collection	0.020	0.426	0.200–0.908
DJ/GJ leak	0.041	0.307	0.099–0.0951

PPH: post pancreatectomy hemorrhage; PEA: pancreaticoenteric anastamosis; HJ: hepaticojejunostomy; DJ: duodenojejunostomy; GJ: gastrojejunostomy; DGE: delayed gastric emptying; ARF: acute renal failure.

**Table 4:** Indications for late reoperations.

Indications	Number of patients	Surgery performed	Interval between PD and re-operation
Group 1			
Incisional hernia	4	Mesh hernioplasty	8–68 months
Pancreatico-jejunostomy stricture	2	Revision PJ/PG	31/36 months
Adhesive SAIO	2	Band release	16/96 months
HJ stricture	1	Revision HJ	29 months
Persistent gastroparesis	1	Distal gastrectomy	26 months
Enterocutaneous fistula (ECF)	1	Repair of ECF	8 months
Afferent limb perforation with intraabdominal collection	1	Abscess drainage, external drainage of afferent limb perforation, lavage and FJ	21 months

Group 2			
Peritoneal dissemination with SAIO	4	Peritoneal nodule biopsy: 2 Jejunojejunal by pass: 2	5–19 months
Liver metastasis			
(i) Metastatic GIST-2	3	Nonanatomical resection: 1 Right hepatectomy: 1	12/30 months
(ii) Ruptured liver metastasis-1		Lavage and drainage: 1	9 months
Scar site recurrence	1	Wide local excision with mesh repair	16 months

Group 3			
Radiation enteritis, jejunal stricture with SAIO	1	Jejuno-jejunal by pass	9 months
Colonic and afferent loop necrosis	1	Excision of afferent loop, right hemicolectomy, revision roux-en-Y hepaticojejunostomy	21 months
DJ stricture	1	Gastrojejunostomy	128 months

Group 4			
Vascular ectasia of jejunum with upper gastrointestinal bleed	1	Partial gastrectomy, revision gastrojejunostomy, side to side jejunojejunostomy	40 months

SAIO: sub-acute intestinal obstruction; GIST: gastrointestinal stromal tumour; PJ: pancreatico-jejunostomy; PG: pancreatico-gastrostomy; HJ: hepatico-jejunostomy; DJ: duodeno-jejunostomy; FJ: feeding jejunostomy.
